# Mitochondrial Genome of *Paraleyrodes minei* Iaccarino (Hemiptera: Aleyrodidae): A New Sugarcane Pest and Phylogenetic Analysis of Aleyrodidae

**DOI:** 10.3390/biology15120968

**Published:** 2026-06-20

**Authors:** Jiong Yin, Changmi Wang, Yinhu Li, Jie Li, Rongyue Zhang, Xiaoyan Wang, Zhiming Luo, Hongli Shan

**Affiliations:** Yunnan Key Laboratory of Sugarcane Genetic Improvement, Yunnan Engineering Research Center of Sugar Industry, Sugarcane Research Institute, Yunnan Academy of Agricultural Sciences, Kaiyuan 661699, China; yinjiong@126.com (J.Y.);

**Keywords:** hemiptera, aleyrodidae, *Paraleyrodes minei*, high-throughput sequencing, mitochondrial genome, phylogeny

## Abstract

*Paraleyrodes minei* is an invasive alien species in China, representing a new record for Yunnan Province and a new sugarcane pest. The mitochondrial genome of *P. minei* was sequenced using the Illumina NovaSeq 6000 sequencing platform. The genome sequence was assembled and annotated, and its structural characteristics and nucleotide composition were analyzed. A phylogenetic tree of 18 species in the family Aleyrodidae was constructed using maximum likelihood (ML) and Bayesian inference (BI) methods to analyze the phylogenetic relationship of *P. minei* within the family Aleyrodidae. The results indicated that the mitochondrial genome of *P. minei* was 18,774 bp in length and contained 13 protein-coding genes (PCGs), 22 transfer RNA (tRNA) genes, 2 ribosomal RNA (rRNA) genes, and 1 non-coding control region. The A+T content of the mitochondrial genome of *P. minei* was 80.93%, indicating a marked A+T preference. ATN was used as the start codon for the PCGs, and TAA, TAG, TA, and T were used as the stop codons. In the secondary structure of tRNA, the TΨC arm was missing in *trnA*, *trnC*, and *trnG*, and the DHU arm was missing in *trnS1* and *trnS2*, with G-U base mismatches present. The phylogenetic tree revealed that the 18 species of 10 genera in the two subfamilies of the family Aleyrodidae clustered into two major branches: the subfamilies Aleyrodinae and Aleurodicinae. All 10 genera were monophyletic groups; among them, the genus *Paraleyrodes* and the genus *Aleurodicus* formed a sister relationship, and both belonged to the subfamily Aleurodicinae. This study represents the first successful sequencing of the mitochondrial genome of *P. minei*, as well as the first mitochondrial genome of the genus *Paraleyrodes*, laying the foundation for the control of *P. minei* and the analysis of phylogenetic relationships among various genera of the family Aleyrodidae.

## Simple Summary

*Paraleyrodes minei*, a new pest of sugarcane, primarily sucks sugarcane sap by congregating in groups of adults and nymphs on the undersides of sugarcane leaves, causing sugarcane leaves to show yellowish-white patches and impairing photosynthesis. The mitochondrial genome of *P. minei* is the first mitochondrial genome of the genus *Paraleyrodes*. These data can not only enrich information on the mitochondrial genomes of insects in the family Aleyrodidae but also provide new ideas for an in-depth understanding of the evolutionary relationships of these insects.

## 1. Introduction

Mitochondria are organelles with independent genetic material in eukaryotic cells. They are the sites where cells produce energy and are involved in processes such as cell differentiation, cellular information transmission, and apoptosis [1,2]. The insect mitochondrial genome is a closed, double-stranded circular molecule and 15 to 20 kb in size that usually contains 13 protein-coding genes (PCGs), 2 ribosomal RNA (rRNA) genes, 22 transfer RNA (tRNA) genes, and one or more non-coding regions (NCRs) containing transcriptional and replication signals [3]. As a molecular genetic marker, the insect mitochondrial genome has been widely used in studies involving rapid insect identification, species evolution, phylogenetics, and population genetics because of its simple structure, stable composition, rapid evolutionary rate, high copy number, maternal inheritance, and low recombination rate [4,5,6].

Whitefly belongs to the family Aleyrodidae under the suborder Sternorrhyncha of the order Hemiptera. It is a type of tiny herbivorous sucking insect that mainly causes damage by sucking the sap of host plants with its nymphs and adults. Many species of whiteflies are important agricultural and forestry pests. Some species are also vectors of many plant virus diseases and can induce the occurrence of sooty mold disease [7,8]. Currently, more than 1631 species of whiteflies have been recorded worldwide, belonging to 166 genera and three subfamilies. The three subfamilies are Udamosolinae, Aleurodicinae, and Aleyrodinae. Among them, there are two subfamilies, 50 genera, and 258 species of whiteflies recorded in China [9].

*Paraleyrodes minei* Iaccarino, 1989, also known as the nesting whitefly, belongs to the order Hemiptera, suborder Sternorrhyncha, family Aleyrodidae, subfamily Aleurodicinae, and genus *Paraleyrodes* [10,11,12]. Native to South America and first recorded on citrus in Syria, this whitefly has now spread to 30 countries across the Americas, Europe, Asia, and Africa [12,13,14,15,16,17,18]. It feeds primarily on woody plants, with 132 species from 51 families serving as host plants [11,14,17,19,20,21,22,23,24,25,26,27,28]. As an invasive alien species in China, *P. minei* has now spread to Hong Kong and Hainan [29]. This is the first report from Yunnan, representing a new record for the province. It also marks the first documented instance of the species feeding on sugarcane, establishing it as a new pest record for sugarcane [30]. As adults and nymphs, *P. minei* mainly infest the undersides of sugarcane leaves, sucking the plant sap. Occasionally, they can also be found on the upper surfaces of the leaves. The affected areas of the leaves show chlorotic yellowish-white spots, which expand into streaks as the damage intensifies, affecting photosynthesis. Currently, researchers have conducted only preliminary studies on the morphological characteristics, occurrence, damage, and biological and ecological features of *P. minei* [12,24,26,31,32]. In a previous study, the author identified *P. minei* using morphological observation and mitochondrial cytochrome c oxidase subunit I (COI) sequence analysis methods [30]. There are no studies on the complete mitochondrial genome of *P. minei*, and little is known about its genetic information and its phylogenetic relationship with other species in the family Aleyrodidae. Sequencing the complete mitochondrial genome of *P. minei* can not only provide information on the mitochondrial genomes of insects in the family Aleyrodidae but also provide new insights into the evolutionary relationships of these insects.

In this study, the first complete mitochondrial genome sequence of *P. minei* was obtained using high-throughput sequencing technology. The structural characteristics of the mitochondrial genome, including nucleotide composition, codon usage frequency, and the secondary structures of tRNA and rRNA, were analyzed. By combining the mitochondrial genome sequences of 18 species of Aleyrodidae that had been published as the ingroup and the mitochondrial genome sequences of two species from Chermidae and Acarina as the outgroup, the internal phylogenetic relationships of Aleyrodidae were constructed using the maximum likelihood (ML) and Bayesian inference (BI) methods. The phylogenetic position of *P. minei* was explored to provide mitochondrial genome data that facilitate a better understanding of the phylogeny of the family Aleyrodidae and *P. minei*.

## 2. Materials and Methods

### 2.1. Insect Sample Collection and DNA Extraction

Adults of *P. minei* were collected from sugarcane in Yongkang Town, Yongde County, Lincang City, Yunnan Province, China (99.375342° E, 24.158125° N), in October 2023. The collected *P. minei* samples were immersed in an anhydrous ethanol solution and stored in a −80 °C ultralow-temperature refrigerator at the Insect Laboratory of the Sugarcane Research Institute, Yunnan Academy of Agricultural Sciences. Total DNA was extracted from 50 samples of *P. minei* using an animal genomic DNA extraction kit (Genepioneer Biotechnologies Co., Ltd., Nanjing, China). The quality and concentration of the total DNA of the samples were determined using a NanoDrop 2000 spectrophotometer (Thermo Fisher Scientific, Waltham, MA, USA) and 1% agarose gel electrophoresis, respectively.

### 2.2. High-Throughput Sequencing and Mitochondrial Genome Assembly

The qualified DNA samples with a concentration greater than 50 μg were sent to Genepioneer Biotechnologies Co., Ltd. (Nanjing, China) to construct a 350 bp small-fragment library. The constructed small-fragment library was sequenced using the Illumina NovaSeq 6000 sequencing platform (Illumina, USA) with paired-end (PE150) sequencing. The raw data were quality-controlled using Fastp v0.23.4 software (https://github.com/OpenGene/fastp (accessed on 5 May 2024)). Mitochondrial genome assembly and splicing of the clean reads obtained from sequencing were performed using SPAdes v3.10.1 software (http://cab.spbu.ru/software/spades/ (accessed on 5 May 2024)) [33], thereby yielding high-quality mitochondrial genome data for *P. minei*. The mitochondrial genome map of the whitefly was plotted using OGDRAW (https://chlorobox.mpimp-golm.mpg.de/OGDraw.html (accessed on 5 May 2024)) [34].

### 2.3. Mitochondrial Genome Annotation and Analysis

The sequenced mitochondrial genome of *P. minei* was preliminarily annotated using the online tool Mitos2 (2.1.10) (http://mitos2.bioinf.uni-leipzig.de/ (accessed on 5 May 2024)) [35]. The preliminary annotation results were compared with those of closely related species, and after manual correction, the final annotation results were obtained and submitted to NCBI. The secondary structures of 22 tRNAs from *P. minei* were predicted using Mitos2 (http://mitos2.bioinf.uni-leipzig.de/ (accessed on 5 May 2024)). Through sequence alignment and validation with the mitochondrial genomes of closely related species, the gene boundaries of the PCGs, tRNAs, and *rrnL* and *rrnS* genes in the mitochondrial genome were manually corrected, and the complete annotated mitochondrial genome sequence was uploaded to GenBank. The nucleotide composition and codon usage frequency of all the PCGs in the mitochondrial genome of *P. minei* were analyzed using MEGA X (12.1) software (accessed on 5 May 2024) [36]. Nucleotide composition bias was calculated using the formulas $AT\text{-}skew=\left(A-T\right)/\left(A+T\right)$ and $GC\text{-}skew=\left(G-C\right)/\left(G+C\right)$.

### 2.4. Analysis of Phylogenetic

The complete mitochondrial genome sequences of 18 species from the family Aleyrodidae were downloaded from the GenBank database. The Asian citrus psyllid, *Diaphorina citri*, from the subfamily Diaphorininae of the family Chermidae, was used as the outgroup. The phylogenetic tree of the family Aleyrodidae was constructed using ML and BI (Table 1). ML phylogenetic analysis was performed using RAxML v8.2.10 (https://cme.h-its.org/exelixis/software.html (accessed on 5 May 2024)). The GTRGAMMA model was selected, and bootstrap analysis (1000 repetitions) was used to test the confidence levels of nodes at each branch of the phylogenetic tree, resulting in the construction of an ML phylogenetic tree. BI phylogenetic analysis was performed using MrBayes v3.2.7a software (http://nbisweden.github.io/MrBayes/ (accessed on 5 May 2024)). The Markov chain Monte Carlo (MCMC) method was employed, with 1,000,000 generations run simultaneously, sampling every 100 generations, and discarding the oldest 25% of the trees to construct a BI phylogenetic tree.

## 3. Results

### 3.1. Structural Characteristics of the Mitochondrial Genome of P. minei

**Figure 1:**
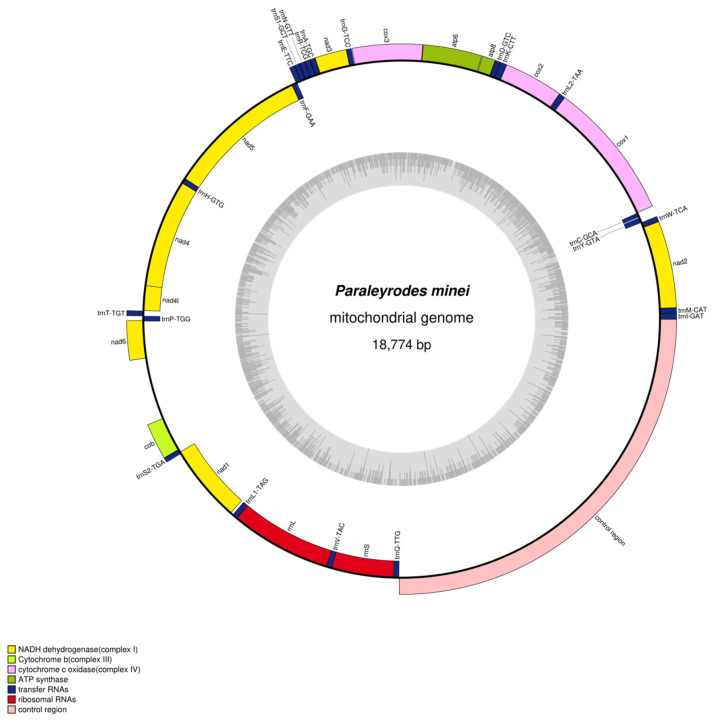
Mitochondrial genome structure map of *Paraleyrodes minei.* The forward-translated genes are located on the outer side of the circle, and the reverse-translated genes are located on the inner side of the circle. The inner ring indicates GC content.

The mitochondrial genome of *P. minei* is 18,774 bp in length, with the GenBank accession number PP727237. It consists of 37 genes, including 13 protein-coding genes (PCGs), 22 tRNA genes, and 2 rRNA genes (*rrnL* and *rrnS*), and it has a closed, double-stranded circular DNA molecular structure (Figure 1). Generally, the strand containing the majority of coding genes is defined as the major coding strand (J-strand), while the other strand is defined as the minor coding strand (N-strand). The J-strand of the *P. minei* mitochondrial genome encodes 23 genes, including 9 PCGs (*nad2*, *cox1*, *cox2*, *atp8*, *atp6*, *cox3*, *nad3*, *nad6*, and *cob*) and 14 tRNA genes (*trnI*, *trnM*, *trnW*, *trnL2*, *trnK*, *trnD*, *trnG*, *trnA*, *trnR*, *trnN*, *trnS1*, *trnE*, *trnT*, and *trnS2*). The N-strand encodes 14 genes, including 4 PCGs (*nad5*, *nad4*, *nad4l*, and *nad1*), 8 tRNA genes (*trnY*, *trnC*, *trnF*, *trnH*, *trnP*, *trnL1*, *trnV*, and *trnQ*), and 2 rRNA genes (*rrnL* and *rrnS*). Additionally, the J-strand includes 1 non-coding control region.

The 37 genes in the mitochondrial genome of *P. minei* are arranged very compactly, with 19 genes showing no overlaps or gaps. There are 10 gene overlaps totaling 38 bp, with little variation in the length of the overlapping sequences; the longest overlap is between *trnE* and *trnF*, with a length of 12 bp. Additionally, there are 8 gene spacings totaling 439 bp, with the longest spacer sequence located between *cob* and *trnS2* and having a length of 386 bp (Table 2).

### 3.2. Nucleotide Composition of the Mitochondrial Genome of P. minei

The A, T, G, and C contents in the mitochondrial genome of *P. minei* are 37.42%, 43.50%, 12.30%, and 6.78%, respectively, with T being the highest and C being the lowest. The A+T content of the whole genome is 80.93%, and the G+C content is 19.07%, indicating a clear preference for A+T. The A+T contents of the PCGs, tRNA genes, rRNA genes, and non-coding control regions are 77.70%, 86.62%, 85.97%, and 84.40%, respectively, indicating that the base composition of the mitochondrial genome of *P. minei* clearly shows a preference for A+T (Table 3).

The skew statistics of the entire nucleotide chain of *P. minei* reveal that the AT-skew is −0.075, indicating that the content of the A base is slightly lower than that of the T base across the entire mitochondrial genome. The GC-skew is 0.29, indicating that the content of the G base in the genome is significantly greater than that of the C base. The AT-skew of the PCGs is negative, indicating a preference for the T base; the AT-skew of the tRNA genes, rRNA genes, and control regions is positive, indicating a preference for the A base. Furthermore, the GC-skews of the PCGs, tRNA genes, and control regions are positive, indicating a preference for the G bases in these three regions. The GC-skew of rRNA is negative, indicating a preference for the C bases in these regions (Table 3).

### 3.3. Protein-Coding Genes of the Mitochondrial Genome of P. minei

The start codon of all 13 PCGs in the mitochondrial genome of *P. minei* is the typical insect start codon ATN. Among these genes, *nad2*, *nad3*, *nad5*, and *nad6* use ATA as the start codon; *cox1*, *atp6*, *cox3*, *nad4*, *nad4l*, and *cob* use ATG as the start codon; and *cox2*, *atp8*, and *nad1* use ATT as the start codon. Four PCGs (*atp8*, *atp6*, *nad4l*, and *nad6*) in the mitochondrial genome of *P. minei* possess the complete stop codon TAA, whereas *cox3* terminates with the complete stop codon TAG. Additionally, *nad2* and *cob* use the incomplete stop codon TA as the stop codon, whereas the other 6 PCGs (*cox1*, *cox2*, *nad3*, *nad5*, *nad4*, and *nad1*) use the incomplete stop codon T as the stop codon (Table 2).

**Figure 2:**
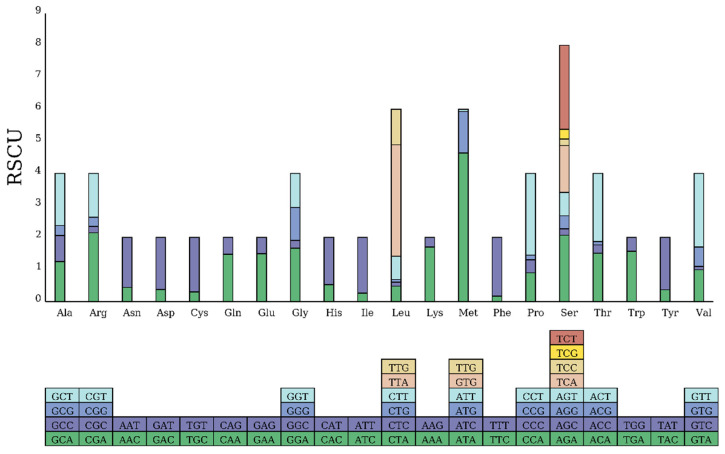
Relative synonymous codon usage (RSCU) of 13 protein-coding genes of *Paraleyrodes minei.* The squares below represent all the codons encoding each type of amino acid, and the height of the columns above indicates the total sum of all the RSCU values of the codons.

The 13 PCGs in the mitochondrial genome of *P. minei* encode a total of 3454 codons (excluding stop codons). The four most frequently used codons are UUU (phenylalanine, Phe), AUU (isoleucine, Ile), UUA (leucine, Leu), and AUA (methionine, Met), with usage frequencies of 486, 328, 298, and 204 times, respectively. The amino acid with the highest percentage in PCGs is Ser (11.77%), followed by leucine (8.82%), methionine (8.82%), and arginine (5.88%) (Figure 2). Analysis of relative synonymous codon usage (RSCU) reveals significant variation in the frequency of codons encoding the same amino acid, suggesting a pronounced bias in codon usage within the mitochondrial genome of the small-nest whitefly.

### 3.4. tRNA and rRNA Genes of the Mitochondrial Genome of P. minei

**Figure 3:**
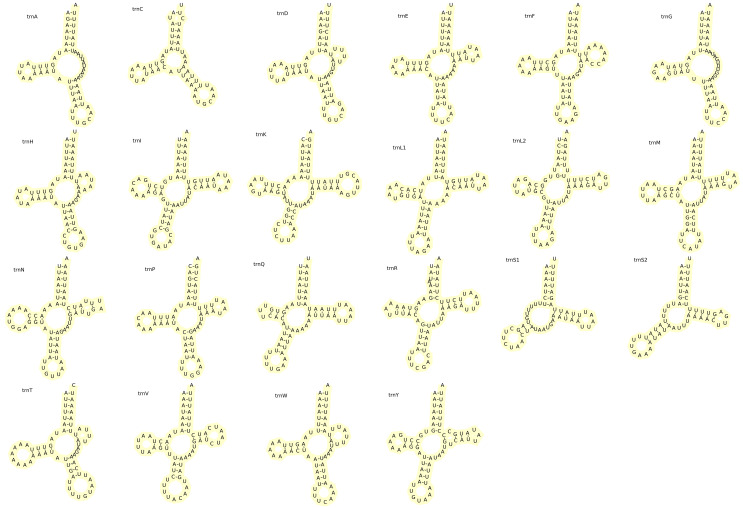
Secondary structure of tRNA genes in the mitochondrial genome of *Paraleyrodes minei*.

The tRNA genes in the mitochondrial genome of *P. minei* are 1354 bp in length, accounting for 7.21% of the mitochondrial genome; individual tRNA gene sequences range from 51 to 70 bp in length. Among the 22 tRNA genes, 17 tRNA genes (*trnD*, *trnE*, *trnF*, *trnH*, *trnI*, *trnK*, *trnL1*, *trnL2*, *trnM*, *trnN*, *trnP*, *trnQ*, *trnR*, *trnT*, *trnV*, *trnW*, and *trnY*) exhibit a typical cloverleaf secondary structure, while the remaining 5 have atypical structures: *trnA*, *trnC*, and *trnG* lack the TΨC arm, and *trnS1* and *trnS2* lack the DHU arm. Twelve pairs of mismatched bases are identified in the secondary structures of the 22 tRNA genes in the mitochondrial genome of *P. minei*. These mismatches are detected in the DHU arms of *trnA*, *trnD*, *trnF*, *trnG*, *trnH*, and *trnT*; the amino acid-accepting arm of *trnA*; the TΨC arm of *trnN;* and the anticodon arms of *trnM* and *trnR*. Specifically, one mismatched base pair each is found in the amino acid-accepting arm and DHU arm of *trnA*, the TΨC arm of *trnG*, and the anticodon arms of *trnM* and *trnR*. Additionally, mismatches are present in the TΨC arm of *trnN* and the antisense codon arms of *trnM* and *trnR*. Among these, the amino acid-accepting arm and DHU arm of *trnA* each contain one mismatched base pair, the DHU arm of *trnG* contains two mismatched base pairs, and the TΨC arm and antisense codon arm of *trnM* each contain one mismatched base pair. All the mismatched bases are G-U (Figure 3).

The mitochondrial genome of *P. minei* contains two rRNA genes, *rrnL* and *rrnS*, whose lengths are 1186 bp and 724 bp, respectively. They are located on the minor coding strand (N-strand) and are separated by *trnV*. The total length of the rRNA genes is 1910 bp, with A, T, C, and G contents of 51.41%, 34.55%, 7.28%, and 6.75%, respectively. The A+T content is 85.97%, which is higher than the A+T content of the entire mitochondrial genome. The AT-skew is positive, and the GC-skew is negative, indicating a preference for A and C bases in *P. minei* (Table 3).

### 3.5. Phylogenetic Analysis

**Figure 4:**
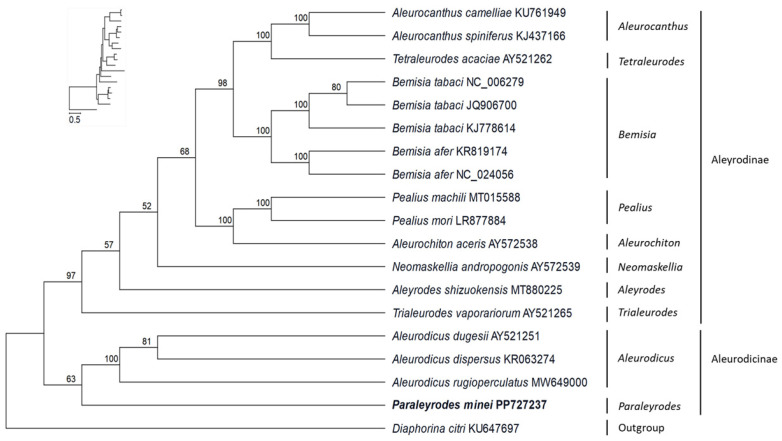
Phylogenetic tree of the nucleotide sequences of 13 protein-coding genes constructed using maximum likelihood.

**Figure 5:**
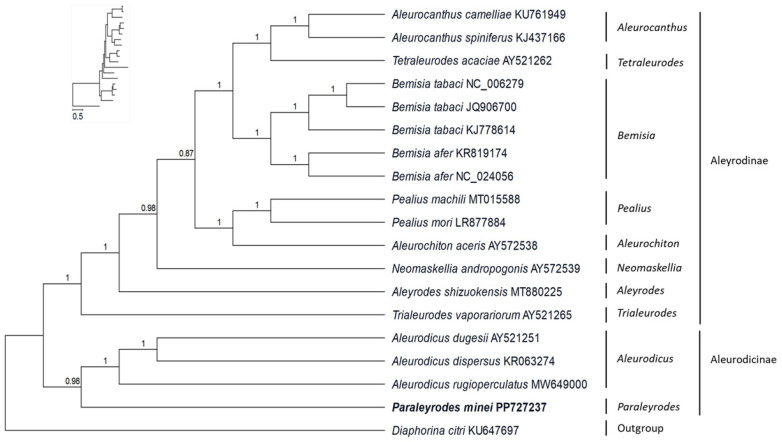
Phylogenetic tree of the nucleotide sequences of 13 protein-coding genes constructed using Bayesian inference methods.

The results of the phylogenetic analysis of the family Aleyroididae based on ML and BI are shown in Figure 4 and Figure 5. The tree topologies constructed by the two phylogenetic analysis methods are essentially consistent. The clustering results show that the 18 species of the family Aleyrodidae are grouped into two major clades: the subfamily Aleyrodinae and the subfamily Aleurodicinae. The first major clade is the subfamily Aleyrodinae, which includes the genera *Aleurocanthus*, *Tetraleurodes*, *Bemisia*, *Pealius*, *Aleurochiton*, *Aleyrodes*, *Trialeurodes*, and *Neomaskellia*. All eight genera are monophyletic. The second major clade is the subfamily Aleurodicinae, which includes the genera *Aleurodicus* and *Paraleyrodes*. Both genera are monophyletic and form a sister group relationship. Among these, *Aleurodicus rugioperculatus*, *Aleurodicus dugesii*, and *Aleurodicus dispersus* form a single clade within the genus *Aleurodicus*, whereas *P. minei* serves as the representative of the genus *Paraleyrodes*, forming a separate clade.

### 3.6. Gene Rearrangement

The mitochondrial gene arrangement in insects is relatively conserved. The mitochondrial gene arrangement of *Drosophila yakuba* is considered to be the gene arrangement of ancestral arthropods [37,38]. On the basis of changes in gene positions during rearrangement, these can be classified as shuffling, translocation, and inversion [39]. Mitochondrial gene rearrangement is a common phenomenon among all species of the family Aleyrodidae. Like most whiteflies, the mitochondrial genome of *P. minei* also exhibits gene rearrangements (Figure 1); however, the number of rearranged genes is limited. Specifically, shuffling occurred in *trnY* and *trnC*, whereas translocations occurred in *trnS1*, *trnS2*, and *trnQ*. All these rearrangements occurred in tRNA genes and were primarily localized at the *nad3*–*cox3* locus.

## 4. Discussion

This study represents the first determination of the complete mitochondrial genome sequence of the new sugarcane pest *P. minei*. The mitochondrial genome comprises 13 protein-coding genes (PCGs), 22 tRNA genes, 2 rRNA genes, and 1 non-coding control region, reflecting the typical structural characteristics of insect mitochondrial genomes [2]. The A+T content of the whole genome is 80.93%, indicating a significant A+T bias, which is consistent with the mitochondrial genome characteristics of previously reported species in the family Aleyrodidae [40]. The start codons of all the PCGs are ATN, whereas the stop codons include both complete types (TAA and TAG) and incomplete types (TA and T), which is consistent with the general characteristics of insect mitochondrial genomes [41]. Secondary structure analysis of tRNAs revealed that *trnA*, *trnC*, and *trnG* lack the TΨC arm, whereas *trnS1* and *trnS2* lack the DHU arm and exhibit G-U mismatches. Such structural variations have been reported in the family Aleyrodidae and other Hemiptera insects and may be related to evolutionary adaptations of the mitochondrial genome [42]. Gene arrangement analysis revealed that *P. minei* has a rearrangement of tRNA genes, primarily occurring at the *nad3*–*cox3* locus. These findings are consistent with results from previous studies [42,43]. This provides a new case for exploring the evolutionary patterns of mitochondrial genomes in the family Aphididae and even the suborder Heteroptera [39]. This unique gene rearrangement pattern, involving changes in the positions of protein-coding genes, may have evolved from an ancestral gene arrangement following a random loss event during tandem duplication [44].

Phylogenetic trees constructed using ML and BI based on the nucleotide sequences of 13 PCGs show consistent topologies, dividing the 18 species of the family Aleyrodidae into two major clades: the subfamily Aleyrodinae and the subfamily Aleurodicinae. Each genus is a monophyletic group, supporting the existing taxonomic framework of the family Aphrophor [10].

Phylogenetic analysis revealed that the genus *Paraleyrodes*, to which the species *P. minei* belongs, forms a sister group relationship with the genus *Aleurodicus*, and both belong to the subfamily Aleurodicinae. These results confirm the taxonomic position of *P. minei* within this subfamily from a molecular systematics perspective and provide genomic-level evidence for its evolutionary relationship with closely related genera [45]. Studies analyzing the association between selection pressure and genetic rearrangements have indicated that there is a correlation between the evolutionary rates of mitochondrial genes and rearrangement events across different subfamilies of the family Aleyrodidae [42]. The tRNA gene rearrangement observed in *P. minei* in this study may provide clues for further exploration of genomic evolutionary mechanisms within the subfamily Aleurodicinae.

In this study, the complete mitochondrial genome sequence of *P. minei* was obtained, and the first mitochondrial genome sequence from the genus *Paraleyrodes* was reported. These data not only enrich the molecular genetic information of the family Aleyrodidae but also provide a crucial foundation for subsequent molecular phylogenetic studies at higher taxonomic levels within the family (e.g., between subfamilies and genera). Furthermore, the analysis of the mitochondrial genome of *P. minei*, a newly invasive sugarcane pest in Yunnan, China, can aid in the development of specific molecular markers for this species. These markers can be applied to rapid field identification, tracing of invasion pathways, and monitoring of population genetic structure, thereby providing technical support for the formulation of scientific monitoring, early warning, and control strategies [31]. Furthermore, regarding the invasive species *P. minei*, future studies could utilize mitochondrial genome data from this research to investigate the genetic diversity and dispersal dynamics of different geographic populations of *P. minei*, thereby providing a more comprehensive assessment of its invasive ecological risks.

## 5. Conclusions

We sequenced the mitochondrial genome of the new sugarcane pest *P. minei*. This mitochondrial genome is a typical closed circular, double-stranded DNA molecule, 18,774 bp in length, with a significant A+T bias. The 13 PCGs begin with ATN and end with TAA, TAG, TA, and T as stop codons. Among the 22 tRNA genes, 17 possess a typical cloverleaf secondary structure, whereas the remaining five exhibit atypical structures; three of these (*trnA*, *trnC*, and *trnG*) lack the TΨC arm, and two (*trnS1* and *trnS2*) lack the DHU arm. Using ML and BI, we inferred the phylogenetic relationships among 18 species of whiteflies, *Diaphorina citri*, and *Tetranychus urticae*. The two major clades, the subfamily Aleyrodinae and the subfamily Aleurodicinae, comprise 10 genera, all of which are monophyletic groups; *Paraleyrodes* and *Aleurodicus* form a sister group.

## Figures and Tables

**Table 1:** Taxonomic information and GenBank accession numbers of all species used in phylogenetic analysis.

Group	Subfamily	Genus	Species	Accession Number	Length (bp)
Ingroup	Aleurodicinae	*Paraleyrodes*	*Paraleyrodes minei*	PP727237	18,774
	Aleurodicinae	*Aleurodicus*	*Aleurodicus dugesii*	AY521251	15,723
	Aleurodicinae	*Aleurodicus*	*Aleurodicus dispersus*	KR063274	16,170
	Aleurodicinae	*Aleurodicus*	*Aleurodicus rugioperculatus*	MW649000	15,060
	Aleyrodinae	*Aleurocanthus*	*Aleurocanthus camelliae*	KU761949	15,188
	Aleyrodinae	*Aleurocanthus*	*Aleurocanthus spiniferus*	KJ437166	15,220
	Aleyrodinae	*Aleurochiton*	*Aleurochiton aceris*	AY572538	15,388
	Aleyrodinae	*Aleyrodes*	*Aleyrodes shizuokensis*	MT880225	16,687
	Aleyrodinae	*Bemisia*	*Bemisia tabaci Asia*	KJ778614	15,210
	Aleyrodinae	*Bemisia*	*Bemisia tabaci MED*	JQ906700	15,632
	Aleyrodinae	*Bemisia*	*Bemisia tabaci New World*	NC_006279	15,322
	Aleyrodinae	*Bemisia*	*Bemisia afer Africa*	NC_024056	14,968
	Aleyrodinae	*Bemisia*	*Bemisia afer China*	KR819174	15,300
	Aleyrodinae	*Neomaskellia*	*Neomaskellia andropogonis*	AY572539	14,496
	Aleyrodinae	*Pealius*	*Pealius machili*	MT015588	15,736
	Aleyrodinae	*Pealius*	*Pealius mori*	LR877884	15,101
	Aleyrodinae	*Tetraleurodes*	*Tetraleurodes acaciae*	AY521262	15,080
	Aleyrodinae	*Trialeurodes*	*Trialeurodes vaporariorum*	AY521265	18,414
Outgroup	Diaphorininae	*Diaphorina*	*Diaphorina citri*	KU647697	14,996

**Table 2:** Annotation of the mitochondrial genome of *Paraleyrodes minei*.

Gene	Strand	Location	Size (bp)	Anticodon	Start Codon	Stop Codon	Intergenic Length
*trnI*	+	1–67	67	GAT	-	-	0
*trnM*	+	64–128	65	CAT	-	-	−4
*nad2*	+	144–1084	941	-	ATA	TA-	15
*trnW*	+	1085–1144	60	TCA	-	-	0
*trnY*	-	1143–1205	63	GTA	-	-	−2
*trnC*	-	1214–1264	51	GCA	-	-	8
*cox1*	+	1263–2799	1537	-	ATG	T--	−2
*trnL2*	+	2800–2863	64	TAA	-	-	0
*cox2*	+	2864–3524	661	-	ATT	T--	0
*trnK*	+	3525–3594	70	CTT	-	-	0
*trnD*	+	3595–3653	59	GTC	-	-	0
*atp8*	+	3654–3803	150	-	ATT	TAA	0
*atp6*	+	3808–4461	654	-	ATG	TAA	4
*cox3*	+	4462–5238	777	-	ATG	TAG	0
*trnG*	+	5243–5300	58	TCC	-	-	4
*nad3*	+	5298–5652	355	-	ATA	T--	−3
*trnA*	+	5653–5710	58	TGC	-	-	0
*trnR*	+	5709–5771	63	TCG	-	-	−2
*trnN*	+	5772–5837	66	GTT	-	-	0
*trnS1*	+	5835–5889	55	GCT	-	-	−3
*trnE*	+	5890–5951	62	TTC	-	-	0
*trnF*	-	5940–6002	63	GAA	-	-	−12
*nad5*	-	6003–7668	1666	-	ATA	T--	0
*trnH*	-	7669–7727	59	GTG	-	-	0
*nad4*	-	7728–9000	1273	-	ATG	T--	0
*nad4l*	-	8994–9290	297	-	ATG	TAA	−7
*trnT*	+	9292–9352	61	TGT	-	-	1
*trnP*	-	9351–9413	63	TGG	-	-	−2
*nad6*	+	9415–9840	426	-	ATA	TAA	1
*cob*	+	9840–10,582	743	-	ATG	TA-	−1
*trnS2*	+	10,969–11,022	54	TGA	-	-	386
*nad1*	-	11,043–11,949	907	-	ATT	T--	20
*trnL1*	-	11,950–12,014	65	TAG	-	-	0
*rrnL*	-	12,015–13,200	1186	-	-	-	0
*trnV*	-	13,201–13,263	63	TAC	-	-	0
*rrnS*	-	13,264–13,987	724	-	-	-	0
*trnQ*	-	13,988–14,051	64	TTG	-	-	0
Control region	+	14,052–18,774	4723	-	-	-	0

**Table 3:** Nucleotide composition of the mitochondrial genome of *Paraleyrodes minei*.

Gene Sequences	Size (bp)	A%	T%	G%	C%	A+T%	G+C%	AT-Skew	GC-Skew
Whole mitogenome	18,774	37.42	43.50	12.30	6.78	80.93	19.07	−0.075	0.29
PCGs	10,387	30.62	47.09	12.15	10.15	77.70	22.30	−0.212	0.09
tRNAs	1353	44.42	42.20	7.91	5.47	86.62	13.38	0.026	0.182
rRNAs	1910	51.41	34.55	6.75	7.28	85.97	14.03	0.196	−0.037
Control region	4723	51.05	33.35	9.15	6.46	84.40	15.60	0.21	0.172

## Data Availability

Data are contained within the article.
